# Combinations of Legume Protein Hydrolysates Synergistically Inhibit Biological Markers Associated with Adipogenesis

**DOI:** 10.3390/foods9111678

**Published:** 2020-11-17

**Authors:** Cecilia Moreno, Luis Mojica, Elvira González de Mejía, Rosa María Camacho Ruiz, Diego A. Luna-Vital

**Affiliations:** 1 Tecnología de Alimentos, Centro de Investigación y Asistencia en Tecnología y Diseño del Estado de Jalisco, A.C., Guadalajara 44270, Mexico; cemoreno_al@ciatej.edu.mx (C.M.); lmojica@ciatej.mx (L.M.); 2 Department of Food Science and Human Nutrition, University of Illinois at Urbana-Champaign, Champaign, IL 61801, USA; edemejia@illinois.edu; 3 Biotecnología Industrial, Centro de Investigación y Asistencia en Tecnología y Diseño del Estado de Jalisco, A.C., Guadalajara 44270, Mexico; rcamacho@ciatej.net.mx; 4 Tecnologico de Monterrey, School of Engineering and Science, Ave. Eugenio Garza Sada 2501, Monterrey 64849, N.L., Mexico

**Keywords:** legumes, protein hydrolysates, anti-adipogenic potential, antioxidant capacity, peptide synergism

## Abstract

The objective was to investigate the anti-adipogenesis potential of selected legume protein hydrolysates (LPH) and combinations using biochemical assays and in silico predictions. Black bean, green pea, chickpea, lentil and fava bean protein isolates were hydrolyzed using alcalase (A) or pepsin/pancreatin (PP). The degree of hydrolysis ranged from 15.5% to 35.5% for A-LPH and PP-LPH, respectively. Antioxidant capacities ranged for ABTS^•+^ IC_50_ from 0.3 to 0.9 Trolox equivalents (TE) mg/mL, DPPH^•^ IC_50_ from 0.7 to 13.5 TE mg/mL and nitric oxide (NO) inhibition IC_50_ from 0.3 to 1.3 mg/mL. LPH from PP–green pea, A–green pea and A–black bean inhibited pancreatic lipase (PL) (IC_50_ = 0.9 mg/mL, 2.2 mg/mL and 1.2 mg/mL, respectively) (*p* < 0.05). For HMG-CoA reductase (HMGR) inhibition, the LPH from A–chickpea (0.15 mg/mL), PP–lentil (1.2 mg/mL), A–green pea (1.4 mg/mL) and PP–green pea (1.5 mg/mL) were potent inhibitors. Combinations of PP–green pea + A–black bean (IC_50_ = 0.4 mg/mL), A–green pea + PP–green pea (IC_50_ = 0.9 mg/mL) and A–black bean + A–green pea (IC_50_ = 0.6 mg/mL) presented synergistic effects to inhibit PL. A–chickpea + PP–lentil (IC_50_ = 0.8 mg/mL) and PP–lentil + A–green pea (IC_50_ = 1.3 mg/mL) interacted additively to inhibit HMGR and synergistically in the combination of A–chickpea + PP–black bean (IC_50_ = 1.3 mg/mL) to block HMGR. Peptides FEDGLV and PYGVPVGVR inhibited PL and HMGR in silico, showing predicted binding energy interactions of −7.6 and −8.8 kcal/mol, respectively. Combinations of LPH from different legume protein sources could increase synergistically their anti-adipogenic potential.

## 1. Introduction

Obesity is defined as excessive fat accumulation, characterized by increased visceral white adipose tissue mass and abnormalities in lipid metabolism that present a risk to health [1,2]. However, its prevalence has doubled around the world and steadily increased over the past 50 years, reaching pandemic levels [3]. This pathological condition results from complex interactions between genes and environmental factors, such as calorie-dense food intake, sedentary lifestyle and stress [4,5].

Adipose cells store energy in the form of triglycerides as well as controlling lipid mobilization and its distribution in the body [6,7,8]. As a result of increased fat storage, excessive adipose tissue expansion alters its histology and function. Consequently, interactions among adipocytes and immune cells at different stages of this process trigger adipocyte lipolysis, increasing circulating free fatty acids, as well as the production of multiple proinflammatory factors [9].

Several pharmacological agents have been developed to influence eating behavior, food intake, energy expenditure and nutrient absorption [10]. Currently, lorcaserin, phentermine/topiramate, naltrexone/bupropion, liraglutide and orlistat are anti-obesity drugs that have been approved by the U.S. Food and Drug Administration (FDA) [11]. For instance, tetrahydrolipstatin (orlistat) is marketed for the long-term regulation of energy intake; this works by inhibiting pancreatic lipase, an enzyme that breaks down triglycerides in the intestinal lumen. Once this enzyme is inactivated, it is unable to hydrolyze fats into fatty acids and monoglycerides, leading to their elimination through the feces [12].

Statins are another class of drugs prescribed as a lipid-lowering medication. Their mechanism of action is through the inhibition of the enzyme 3-hydroxy-3-methylglutaryl coenzyme A (HMG-CoA) reductase (HMGR). This enzyme catalyzes the rate-limiting step of the mevalonate pathway that leads to cholesterol biosynthesis. Inhibition of its enzymatic activity lowers endogenous cholesterol and serum low-density lipoprotein levels [13].

Pharmacological management of obesity has been unsuccessful due to adverse side effects generating safety concerns. For example, the use of orlistat has been associated with several gastrointestinal adverse effects, such as oily stools, diarrhea, abdominal pain and fecal spotting, and even a few cases of serious hepatic adverse effects [14]. Statins have also shown concerning side effects including myositis, myalgia, rhabdomyolysis muscle pain, fatigue and weakness [15].

Legumes are an excellent source of proteins and, when they are enzymatically digested, they become peptides with multiple sizes that can exert a wide spectrum of biological potentials [16,17,18]. Hence, the incorporation of bioactive compounds from legumes into diets could exert beneficial effects by regulating lipid metabolism, resulting in a potential alternative in the prevention or as an adjuvant in the treatment of obesity [19,20]. Furthermore, studies with legume-derived hydrolysates have shown beneficial effects on immunity, inflammation, infection, hypertension, hypercholesterolemia, type 2 diabetes and some types of cancer [21,22,23].

Therefore, this study aimed to evaluate the anti-adipogenic potential and antioxidant properties of selected legume protein hydrolysates by comparing their potential to inhibit pancreatic lipase and HMG-CoA reductase using biochemical assays and in silico approaches. Synergistic, additive and antagonistic effects between the combinations of legume protein hydrolysates were also evaluated through isobolographic analyses.

## 2. Materials and Methods

### 2.1. Materials

Raw varieties of *Phaseolus vulgaris* L., *Pisum sativum* L., *Cicer arietinum* L., *Lens culinaris* L. and *Vicia faba* L. were obtained from local farmers of Guadalajara, Mexico. The dry grains were stored at 4 °C until use. Commercial proteases alcalase (EC 3.4.21.62), porcine pepsin (EC 3.4.23.1) and pancreatin (8xUSP, 232-468-9) were purchased from Sigma-Aldrich (St. Louis, MO, USA). DC protein assay was purchased from Bio-Rad Laboratories. Molecular weight protein standard (10 to 250 kDa) and SimplyBlue Safe Stain were purchased from Amersham Pharmacia Biotech (Carlsbad, CA, USA). All other chemicals and reagents were obtained from Sigma-Aldrich (St. Louis, MO, USA).

### 2.2. Legume Protein Isolate Extraction

The extraction of proteins from legumes was conducted using a previously established methodology [24]. Common black bean seeds were soaked in water at room temperature for 16 h. Black bean hulls were manually removed; then, bean cotyledons, green pea, chickpea, lentil and fava bean were ground separately in a commercial blender in a 1:10 grain/water ratio. The pH of the supernatant was adjusted to 8.0 with 1 M sodium hydroxide, and protein extraction was carried out at 35 °C with stirring for 1 h. The mixture was centrifuged at 5000× *g* for 15 min at 25 °C. Then, the pH was adjusted to the isoelectric point of the different legumes with 1 M hydrochloric acid to precipitate proteins, followed by centrifugation at 10,000× *g* for 20 min at 4 °C. The supernatant was discarded and the pellet was freeze-dried in a Lab Conco Freeze Dryer 4.5 (Kansas, MO, USA). Legume protein isolates (LPI) were stored at −20 °C until further analysis.

### 2.3. Protein Hydrolysis

#### 2.3.1. Simulated Gastrointestinal Digestion

Legume protein hydrolysates (LPH) were obtained after simulated gastrointestinal digestion with pepsin/pancreatin (PP) following the method described by Mojica et al. (2015) [25]. Briefly, LPI was suspended in water (1:20 *w*/*v*) and autoclaved for 20 min at 121 °C to denature proteins and improve hydrolysis. Sequential enzyme digestion was carried out with pepsin/substrate 1:20 (*w*/*w*) (pH 2.0) followed by pancreatin/substrate 1:20 (*w*/*w*) at pH 7.5 at 37 °C for 2 h each. The hydrolysis was stopped by heating at 75 °C for 20 min, and the resulting LPI hydrolysates were centrifuged at 20,000× *g* for 15 min at 4 °C. LPH were dialyzed to eliminate salts using a 500 Da molecular weight cutoff membrane and then freeze-dried in a LabConco FreeZone Freeze Dry System. Hydrolysates were stored at −20 °C until further analysis.

#### 2.3.2. Alcalase Enzymatic Digestion

Alcalase (A) was used for the hydrolysis of LPI. First, a portion of LPI was suspended in water (1:20 *w*/*v*) and autoclaved for 20 min at 121 °C. Then, enzymatic digestion was carried out using a protease/substrate ratio of 1:20 (*w*/*w*), time of hydrolysis 2 h, with pH and temperature optimal for alcalase activity (pH 7.0, T: 50 °C, respectively). Protein hydrolysis was stopped by heating at 75 °C for 20 min, and the resulting LPH were centrifuged at 20,000× *g* for 15 min at 4 °C. LPH were dialyzed to eliminate salts using a 500 Da molecular weight cutoff membrane and then freeze-dried in a LabConco FreeZone Freeze Dry System. Hydrolysates were stored at −20 °C until further analysis [24].

### 2.4. Degree of Hydrolysis (DH)

An aliquot of 64 µL of sample solution was placed in 1 mL of 0.2125 M sodium phosphate buffer pH 8.2. A 0.05% TNBS (trinitrobenzene sulfonic acid) solution was sequentially added to light-protected test tubes and then the reaction mixture was placed in a water bath at 50 °C for 30 min. The reaction was stopped by adding 0.1 M sodium sulfite. After cooling for 15 min at room temperature, the absorbance was measured at 420 nm. The total hydrolysis of the sample was carried out using 6 N HCl at 110 °C for 24 h. A calibration curve was produced using leucine (0 to 10 mM) as standard. DH was calculated according to the following equation:

$$\%DH=\frac{h}{h\;total}*100$$

where *h* is the number of free amino groups in the hydrolyzed sample and *h total* is the number of free amino groups after complete hydrolysis of the LPI.

### 2.5. Gel Electrophoresis Analysis SDS–PAGE (Sodium Dodecyl Sulfate–Polyacrylamide Gel Electrophoresis)

The freeze-dried LPI of the five legume types tested and their respective LPH were analyzed by SDS–PAGE, under reducing conditions (1:20 β-mercaptoethanol, β-ME). Precast (4% to 20%) gradient polyacrylamide, Tris–HCl gels were used with a Bio-Rad Criterion Cell under a constant voltage of 200 V for 35 min. Standards (10 to 250 kDa) were used to calculate molecular mass. After staining with Simply Blue Safe Stain overnight, and destaining with water, the gel was visualized using a Chemidoc^TM^ XRS^+^ System (Bio-Rad Laboratories, Inc., Hercules, CA, USA).

### 2.6. Identification and Characterization of Potentially Bioactive Peptides

LPH were analyzed by liquid chromatography–electrospray ionization–mass spectrometry (LC–ESI–MS/MS) using a Q-tof Ultima mass spectrometer (Water, Milford, CT, USA), equipped with an Alliance 2795 HPLC system. Separation of the components was performed by using a mobile phase of Solvent A (95% H_2_O, 5% ACN and 0.1% formic acid) and Solvent B (95% ACN, 5% H_2_O and 0.1% formic acid) using a flow rate of 400 μL min^−1^. The elution was in a linear gradient (0 min, 100% A; 1 min, 100% A; 2 min, 90% A, 10% B; 6 min, 60% A, 40% B; 10 min, 100% B; 12 min, 100% B; 14 min, 100% A; 15 min, 100% A). The temperature was kept at 20 °C during the whole procedure. A splitter with a split ratio of 1:10 was used, where one part was used by the mass spectrometer and ten parts were used for the waste. The Q-tof Ultima mass spectrometer was equipped with a Z-spray ion source. Using the positive ion electrospray mode (+ESI), the analysis on the Q-tof was carried out in V-mode with an instrument resolution between 9000 and 10,000 based on full width at half maximum, with a flow rate of 400 μL min^−1^. The source temperature was set at 80 °C and desolvation temperatures were set at 250 °C. The Q-tof was operated at a capillary voltage of 3.5 kV and a cone voltage of 35 V. The final detector was a microchannel plate with high sensitivity. The MassLynx 4.1 V software (Waters, Milford, CT, USA) was used to control the instruments and to process the data to obtain the highest probability of the peptide’s sequences. Confirmation of peptide sequences in each legume protein was performed using the BLAST^®^ tool (http://www.blast.ncbi.nlm.nih.gov/Blast.cgi, accessed on 15 February 2019).

The potential biological activity of the peptides was predicted by using BIOPEP^®^ database (http://www.uwm.edu.pl/biochemia, accessed on 16 February 2019). Peptide structures were predicted using the PepDraw tool (http://www.tulane.edu/biochem/WW/PepDraw/, accessed on 16 February 2019).

### 2.7. Antioxidant Capacity Assays

#### 2.7.1. ABTS Radical Scavenging Activity

The ABTS^•+^ radical cation was produced by reacting 7 mM 2,2′-azinobis (3-ethyl-benzothiazoline-6-sulfonic acid) diammonium salt (ABTS) with 2.45 mM potassium persulfate. The mixture was left to stand in the dark at room temperature for 20 h before use (overnight). The radical was stable in this form under these conditions for more than 48 h. The ABTS^•+^ solution was diluted with 0.01 M phosphate buffer (pH 7.4) to an absorbance of 0.70 ± 0.02 at 734 nm. The scavenging activity was expressed as IC_50_ values Trolox equivalent per mg of the sample using the following equation y = 411.91x + 8.4207, R^2^ = 0.99.

#### 2.7.2. DPPH Radical Scavenging Activity

Antioxidant activity of legume hydrolysates was determined using DPPH (2,2-diphenyl-1-picrylhydrazyl) free radical scavenging assay. Protected from light, 100 µL of each sample or buffer as control was added to 1.5 mL of methanolic DPPH solution (0.1 mM) (1,1-diphenyl-2-picrylhydrazyl) and stirred by vortex (3000 rpm) for 30 s. After 30 min of incubation, the absorbance of the solution was read at 517 nm. The analytical curve was constructed using 5–205 µg/mL of the Trolox solution to determine TE (y = 0.0016 x − 0.0294; R^2^ = 0.94). The results were expressed as IC_50_ values of Trolox equivalent (TE) per mg of the sample.

#### 2.7.3. Nitric Oxide (NO) Radical Scavenging

Nitric oxide production was estimated by the accumulation of nitrite (NO_2_), a stable product of the nitric oxide (NO) reaction with oxygen in Griess reagent (1% sulfanilamide and 0.1% *N*-1-(naphtha-yl) ethylenediamine dihydrochloride in 2.5% H_3_PO_4_). Sodium nitroprusside (SNP) was used as the NO donor. NO reacts with oxygen to produce nitrate and nitrite. Briefly, SNP (10 mM) in phosphate-buffered saline was mixed with different concentrations of each LPH sample (0.1–5 mg/mL freeze-dried hydrolysate). The samples were incubated at 25 °C. After 120 min, 0.5 mL incubated solution was mixed with 0.5 mL of Griess reagent. Results were expressed as % inhibition related to PBS treatment [26].

### 2.8. Biochemical Analyses to Determine Anti-Adipogenic Potential

#### 2.8.1. Lipase Activity Measurement Using pH Indicator-Based Lipase Assay

Inhibitory pancreatic lipase activity was determined according to the method implemented by Camacho-Ruiz et al. (2015) [27]. Briefly, to measure the formation of free fatty acids upon hydrolysis of TG (4:0) or TG (8:0), each substrate was prepared with one volume of the substrate (50 mM dissolved in tert-butanol), also containing the pH indicator, which was mixed vigorously on a vortex with nine volumes of buffer solution to reach a final substrate concentration of 5 mM. Buffer solution included 2.5 mM 3-Morpholinopropane-1-sulfonic acid (MOPS), 0.5 mM sodium taurodeoxycholate hydrate (NaTDC), 150 mM NaCl, 6 mM CaCl_2_. Then, 20 μL of enzyme solution at appropriate dilution in buffer was added in each microplate well and 100 μL of substrate emulsion was quickly added using an eight-channel pipette. Subsequently, the plate was placed in a microtiter plate scanning spectrophotometer (x-Mark™, Bio-rad, Hercules, CA, USA) and shaken for 5 s before each reading. The decrease in absorbance at a wavelength corresponding to the λmax of the pH indicator was recorded every 30 s at 37 °C. Blanks without enzyme were performed and data were collected at least in triplicate for 15 min.

#### 2.8.2. Hydroxy-3-methylglutaryl Coenzyme a Reductase (HMG-CoA Reductase) Activity Assay

The HMG-CoA reductase assay kit CS-1090 from Sigma-Aldrich (St. Louis, MO, USA) was carried out under conditions recommended by the manufacturer at 37 °C. Statin drug (pravastatin) was employed as a positive control. In summary, aliquots containing NADPH (4 μL), HMG-CoA substrate (12 μL) and a buffer pH 7.4 were placed into a UV compatible 96-well plate. The analyses were initiated by the addition of HMG-CoA reductase (2 μL) in each well and incubated in the presence or absence of pravastatin (250 nM) or 5–0.1 mg/mL hydrolysate concentration of each LPH. The rate of NADPH consumed was monitored by reading the decrease in absorbance at 340 nm by microtiter plate scanning spectrophotometer (x-Mark™, Bio-rad, Hercules, CA, USA). The HMG-CoA-dependent oxidation of NADPH and the inhibition properties of LPH were measured by the absorbance reduction, which is directly proportional to the enzyme activity.

### 2.9. Isobolographic Analysis of PL Inhibitory Activity by LPH

The interactions were validated by isobolographic analysis in which the combinations were comprised of equieffective doses of the individual components. Using the IC_50_ values of each LPH, the additive line was plotted and the equieffective dose was calculated. Subsequently, a dose–response curve of PL inhibition was obtained in a fixed-ratio for the mixture of LPH (1:1) that was based on the IC_50_ values of each protein hydrolysate. The experimental IC_50_ values for the LPH combinations were calculated. In an isobologram, when the protein hydrolysate combination IC_50_ lies on the theoretical IC_50_ add line, then the mixture is considered to be synergistic. An interaction index (γ) was calculated according to the following formula: IC_50_ combination/IC_50_ theoretical. Gamma values around 1 (γ = 1) indicated additive interaction; γ > 1 implied and antagonistic interaction and γ < 1 indicated a synergistic interaction.

### 2.10. Molecular Docking (In Silico Analysis)

The structural mechanism by which the peptides present in LPH interact with the enzymes, pancreatic lipase and HMG-CoA reductase was evaluated by in silico analysis through molecular docking, as described by Mojica et al. (2017) [28]. Molecular docking analysis was performed to predict individual peptide biological potential using DockingServer^®^ [29]. Peptides were designed using the software Instant MarvinSketch (Chem Axon Ltd., Budapest, Hungary). The MMFF94 force field [30] was used for the energy minimization of ligand molecules, peptides, orlistat and pravastatin. Gasteiger partial charges were added to the peptide ligand atoms (peptides). Non-polar hydrogen atoms were merged, and rotatable bonds were defined. Docking calculations were carried out on PL (1LPB) and HMG-CoA reductase (1DQ9) protein crystal structures. Essential hydrogen atoms, Kollman united atom type charges and solvation parameters were added with the aid of AutoDock tools [31]. Affinity maps and spacing were generated using the Autogrid program. AutoDock parameter set- and distance-dependent dielectric functions were used in the calculation of the van der Waals and the electrostatic terms, respectively. Initial position, orientation and torsions of the ligand molecules were set randomly. Each docking experiment was derived from 100 different runs that were set to terminate after a maximum of 250,000 energy evaluations. The population size was set up to 150. During the search, a translational step of 0.2 Å, and quaternions and torsion steps of 5 were applied.

### 2.11. Statistical Analysis

The results were expressed as the mean ± SD of at least two independent experiments with three repetitions each and analyzed through ANOVA. Statistical significance (*p* < 0.05) was determined using Student’s *t*-test for comparing mean pairs and Tukey’s test for multiple mean comparisons using software JMP version 8.0 (SAS Institute, Cary, NC, USA).

## 3. Results

### 3.1. Protein Profile for LPI and LPH by Gel Electrophoresis Analysis

**Figure 1:**
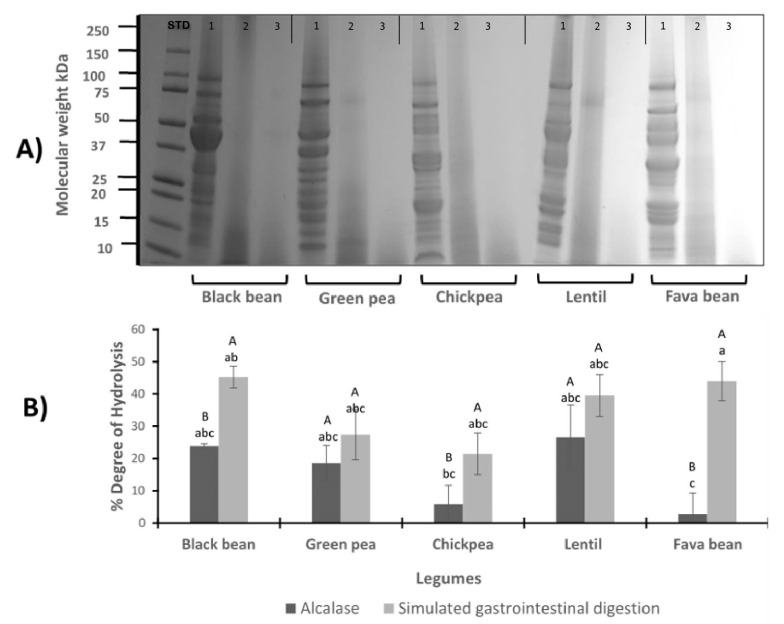
(**A**) Electrophoretic SDS–PAGE profile of legumes. Protein profile of black bean, green pea, chickpea, lentil and fava bean, before and after hydrolysis with alcalase and simulated gastrointestinal digestion. Each sample is presented in 3 wells: 1st well belongs to LPI profile (1), 2nd belongs to protein isolate after hydrolysis with alcalase (2), and the 3rd belongs to protein isolate after pepsin/pancreatin digestion (3). STD: standard. (**B**) Degree of hydrolysis (%) of legume protein isolates hydrolyzed with alcalase (A-LPH) and after simulated gastrointestinal digestion with pp (PP-LPH). Different uppercase letters indicate significant differences between alcalase and pepsin/pancreatin digestion (*p* < 0.05); different lowercase letters indicate significant differences (*p* < 0.05) among legume protein hydrolysates. Results represent the mean ± SD of at least two independent experiments.

The SDS–PAGE analysis was conducted to observe the effect of enzymatic hydrolysis with alcalase and pepsin/pancreatin in the molecular weight distribution of LPI (Figure 1A). Three lanes per legume are depicted. The first lane represents LPI before hydrolysis; the second lane contains the LPI after hydrolysis with the commercial enzyme alcalase (A); and the third lane contains the LPI after simulated gastrointestinal digestion with pepsin and pancreatin (PP). Protein components of the LPI ranged from 10 to 100 kDa. Enzymatic hydrolysis exerted an effect on molecular weight distribution, mostly to the high molecular weight fractions. Black bean protein isolate (first lane) reveals several major bands which are 47 and 44 kDa phaseolin bands [25] (Figure 1A). Phytohemagglutinin is also visible, which corresponds to the 31 kDa band. Major identified proteins in common black bean were phaseolin, lectin, protease and α-amylase inhibitors, Kunitz trypsin inhibitor and Bowman–Birk inhibitor [32]. Green pea LPI revealed convicilin (72 kDa), legumin (25, 39 kDa) and vicilin (44, 32, 16 kDa) fractions [33]. In the case of lentil LPI, the most intense bands observed in the SDS–PAGE correspond to subunits of vicilin (48 kDa) and convicilin (63 kDa). Other lower molecular mass bands were observed which can represent gamma-vicilin and a mixture of albumin polypeptides [34]. The protein profile of chickpea and fava bean was also influenced by the hydrolysis process.

### 3.2. Degree of Hydrolysis

The effect of the digestion conditions on the degree of hydrolysis (DH) was evaluated. Figure 1B shows the comparison between the LPI hydrolyzed with alcalase and by a simulation of gastrointestinal digestion with PP. The average yield of hydrolysis ranged from 2.72% to 26.61% and 21.50% to 45.26% for A-LPH and PP-LPH, respectively. In general, across all the legume hydrolysates, DH showed great variability; however, significant differences between the enzymes alcalase and PP were observed in the black bean, chickpea and fava bean hydrolysates. In the case of green pea and lentil hydrolysates, no statistical differences were found using alcalase and PP enzyme. The lowest values of DH were observed in chickpea and fava bean LPI hydrolyzed with alcalase.

### 3.3. Peptide Sequences and Predicted Bioactivity

The peptide sequences resulting from the PP and alcalase digestion were identified by high-performance–liquid chromatography–electrospray ionization–mass spectrometry (LC–ESI–MS/MS). Twenty-seven peptide sequences were obtained by alcalase enzymatic digestion and thirty-four peptides were sequenced from PP. Peptide identity was confirmed by Blast tool and most peptides were identified in legume parental proteins. Moreover, the physicochemical properties of the peptides are listed in Table 1. A-hydrolysates and PP-hydrolysates showed peptides with around six and eight amino acids, respectively. Most of their amino acids were aliphatic (glycine, proline, valine and alanine), which are non-polar and hydrophobic. Molecular weight for both A and PP peptides ranged from 440 (SPPE) to 1408 Da (VNPDPAGGPTSGRAL). The isoelectric point ranged from the acidic pI 2.78 (DLVLDVPS) to alkaline pI 11.52 (KPSSAAGAVR). The net charge was neutral in 46% of the peptides sequenced; 36% of the peptides presented negative charge and 18% were positively charged. Hydrophobicity ranged from 4.88 (TKAGGTAF) to 22.78 kcal/mol (VELVGPK).

**Figure 2:**
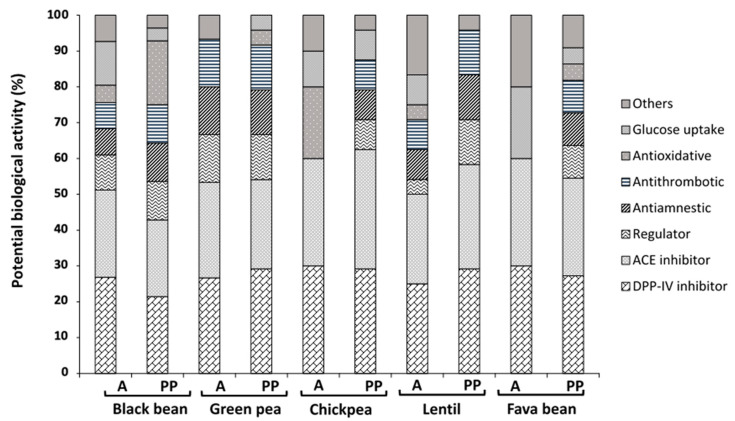
Potential biological activity (%) of different legume-generated peptides using Biopep database. PP, pepsin/pancreatin; A, alcalase; DPP-IV, dipeptidyl peptidase IV inhibitor; ACE, angiotensin-converting enzyme inhibitor; regulator, stomach mucosal membrane activity regulator. Results represent the mean ± SD of at least two independent experiments.

The biological potential of peptide sequences generated by A and PP is shown in Figure 2. The percentage of biological potential is relative to the total bioactive peptides produced by each enzymatic system used. Most peptides in all the legumes evaluated potentially exert activity by blocking dipeptidyl peptidase-IV (DPP-IV) and angiotensin-converting enzyme (ACE). Potential biological activities such as stomach mucosa regulator, antiamnestic, antithrombotic, antioxidative and glucose uptake promoters were also observed.

### 3.4. Antioxidant Capacity

#### 3.4.1. ABTS Radical Scavenging

**Figure 3:**
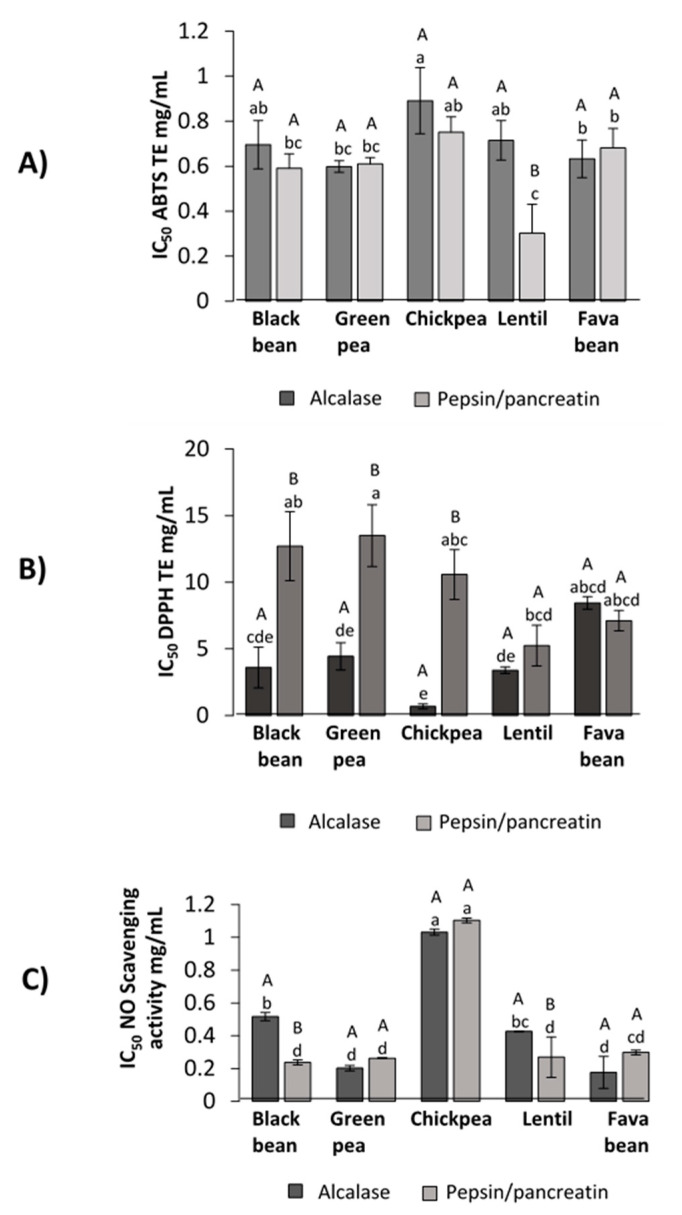
Antioxidant potential of LPH against different radicals. (**A**) ABTS scavenging activity (IC_50_ TE mg/mL), (**B**) DPPH scavenging activity (IC_50_ TE mg/mL), (**C**) nitric oxide scavenging activity (IC_50_ mg/mL). Different capital letters indicate significant differences between alcalase and pepsin/pancreatin digestion (*p* < 0.05); different lowercase letters indicate significant differences (*p* < 0.05) among legume protein hydrolysates. Results represent the mean ± SD of at least two independent experiments.

The antioxidant capacity of the whole hydrolysate (LPH) was measured spectrophotometrically by the disappearance of the blue/green stable ABTS^•+^ radical, caused by scavenging (Figure 3A). The concentration of inhibitor required to produce 50% inhibition was calculated (IC_50_) and the results of ABTS^•+^ radical assay were presented as Trolox equivalent (TE) antioxidant capacity using Trolox as standard. PP–lentil hydrolysate was demonstrated to have the highest ability to scavenge the ABTS^•+^ radical (IC_50_ = 0.30 ± 0.13 TE/mL), which was followed by PP–black bean (IC_50_ = 0.60 ± 0.06 TE/mL) and both PP and A–green pea hydrolysates (IC_50_ = 0.61 ± 0.03 and 0.60 ± 0.03 TE mg/mL, respectively) (*p* < 0.05).

#### 3.4.2. DPPH Inhibition Capacity

The antioxidant activity of LPH measured by DPPH^•^ scavenging capacity is shown in Figure 3B. Hydrolysates obtained from alcalase digestion showed higher DPPH^•^ scavenging activity compared to PP-LPH (*p* < 0.05). As the lower the IC_50_ value, the most potent scavenging capacity, A–chickpea (IC_50_ = 0.68 ± 0.18 mg/mL) had the lowest IC_50_ value, followed by A–lentil (IC_50_ = 3.40 ± 0.30 mg/mL), A–black bean (IC_50_ = 3.60 ± 1.50 mg/mL) and A–green pea (IC_50_ = 4.45 ± 1.03 mg/mL).

#### 3.4.3. Nitric Oxide (NO) Scavenging Capacity

LPH were assessed for NO radical inhibitory activity (Figure 3C). It was found that NO radical inhibitory activity showed no significant differences among IC_50_ values of PP–black bean (0.23 ± 0.01 mg/mL), A and PP–green pea (0.20 ± 0.00 mg/mL and 0.25 ± 0.01 mg/mL, respectively), PP–lentil (0.24 ± 0.04 mg/mL) and A and PP–fava bean (0.16 ± 0.01 mg/mL and 0.28 mg/mL ± 0.01, respectively) hydrolysates.

### 3.5. Anti-Adipogenic Potential

#### 3.5.1. Pancreatic Lipase Inhibitory Activity

**Figure 4:**
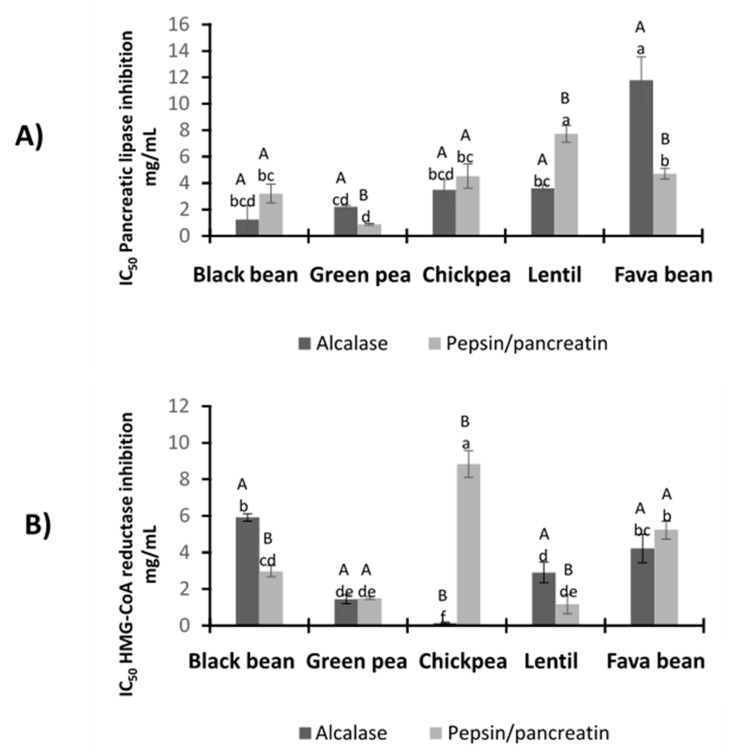
(**A**) Inhibition potential of LPH to pancreatic lipase (IC_50_ mg/mL). Orlistat synthetic inhibitor IC_50_ = 2.73 μg/mL [31]. Different capital letters indicate significant differences between alcalase and pepsin/pancreatin digestion (*p* < 0.05); different lowercase letters indicate significant differences (*p* < 0.05) among legume protein hydrolysates. (**B**) Inhibition potential of LPH to HMG-CoA (IC_50_ mg/mL). Simvastatin synthetic inhibitor IC_50_ = 0.08 μg/mL. Different capital letters indicate significant differences between alcalase and pepsin/pancreatin digestion (*p* < 0.05); different lowercase letters indicate significant differences (*p* < 0.05) among legume protein hydrolysates. Results represent the mean ± SD of at least two independent experiments.

LPH were tested for their ability to inhibit PL. All the hydrolysates demonstrated to inhibit the enzyme in a dose-dependent manner, as shown in Figure 4A. There were no significant differences between PP and A–black bean hydrolysates and PP and A–chickpea, (*p* < 0.05). PP–green pea hydrolysate presented the lowest IC_50_ value (0.8 ± 0.06 mg/mL) among all the legume PP hydrolysates. Nevertheless, the IC_50_ values of the PP–lentil hydrolysate (7.7 ± 0.64 mg/mL) and A–fava bean hydrolysate (11.8 ± 1.75 mg/mL) were significantly higher (less potent inhibition) compared to the rest of the hydrolysates.

#### 3.5.2. HMG-CoA Reductase Inhibitory Activity

HMGR catalyzes the rate-limiting step of the mevalonate pathway that leads to cholesterol biosynthesis; thus, inhibition of its enzymatic activity plays a significant role in lowering endogenous cholesterol levels during hypercholesterolemia [35]. Figure 4B shows the inhibition of HMGR activity by PP and A hydrolysates. A–chickpea was the most effective hydrolysate with an IC_50_ = 0.15 ± 0.04 mg/mL, followed by PP–lentil (IC_50_ = 1.17 ± 0.52 mg/mL), A–green pea (IC_50_ = 1.45 ± 0.25 mg/mL) and PP–green pea (IC_50_ = 1.50 ± 0.07 mg/mL). By contrast, the highest IC_50_ value was observed in PP–chickpea (IC_50_ = 8.84 ± 0.74 mg/mL).

#### 3.5.3. Isobolograms of the LPH Interactions

**Figure 5:**
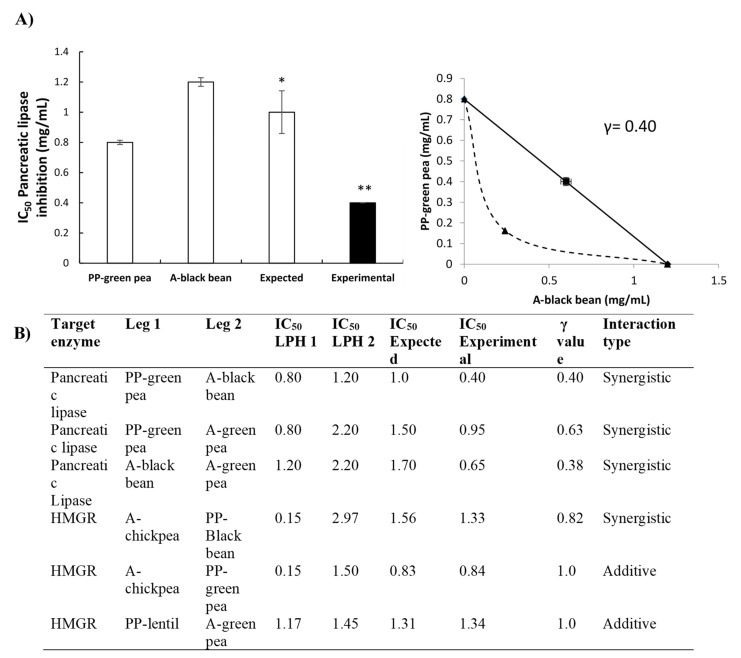
(**A**) Synergistic interaction of PP–green pea and A–black bean hydrolysates to block pancreatic lipase (IC_50_ mg/mL). Bar plots represent the mean ± standard deviation of at least two independent experiments, with only the expected and combined values compared statistically. Different number of stars means significant difference (*p* < 0.05). The lower the values, the more potency. Results represent the mean ± SD of at least two independent experiments. (**B**) Interactions between legume hydrolysate combinations to block pancreatic lipase and 3-hydroxy-3-methylglutaryl coenzyme A reductase HMGR. Results are expressed in IC_50_ mg/mL; γ value < 1 synergism, γ value = 1 additive effect, γ value > 1 antagonism.

The results of the isobolographic PL assay are presented in Figure 5A,B. It was observed that three combinations of LPH showed synergistic potential, namely PP–green pea and A–black bean, PP–green pea and A–green pea, A–black bean and A–green pea, with IC_50_ values of 0.40 ± 0.00 mg/mL, 0.95 ± 0.02 mg/mL and 0.65 ± 0.04 mg/mL, respectively.

For the inhibition of HMGR, interactions among LPH presented synergistic and additive potential (Figure 5B). A synergistic effect was observed with the combination of A–chickpea and PP–black bean (IC_50_ = 1.33 ± 0.07), while additive interactions were observed between A–chickpea and PP–green pea (IC_50_ = 0.84 ± 0.04 mg/mL) and PP–lentil and A–green pea (IC_50_ = 1.34 ± 0.00 mg/mL).

### 3.6. Molecular Docking Study of Peptides Inhibiting Pancreatic Lipase and HMG-CoA Reductase

**Figure 6:**
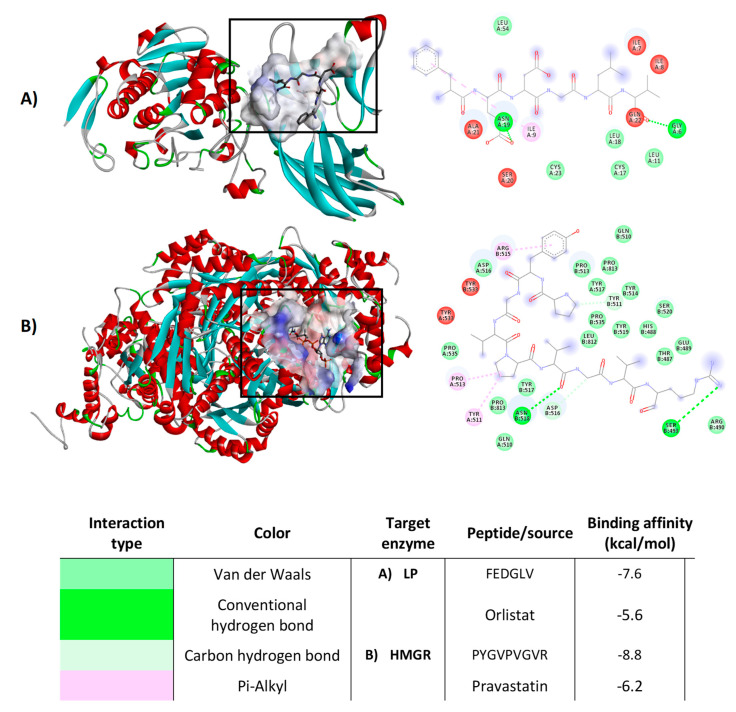
Molecular docking diagrams, examples of the best pose of the most potent legume peptides in the catalytic site of pancreatic lipase (**A**) and HMGR (**B**). Results represent the mean ± SD of at least two independent experiments.

Molecular docking analysis was performed to predict the potential of the LPH to interact with PL and HMG-CoA reductase enzymes. Peptide and macromolecular target docking shows theoretical affinity, type of interactions and distances [36,37]. Table 2 shows the minimum estimated free energy for peptides sequenced from LPH, with PL and HMGR. Estimated free energy indicates that compounds with the most negative value present higher potential to interact with the target enzyme. The peptides studied had free energy values ranging from −5.5 (CSSSSG) to −7.6 (FEDGLV) kcal/mol for PL. Peptide FEDGLV was obtained from A–lentil and CSSSSG from PP–chickpea. In the case of HMGR, binding affinities of peptides ranged from −6.1 (CSSSSG and GPPVDVPQ) to −8.8 (PYGVPVGVR) kcal/mol, obtained from PP–chickpea and PP–fava bean, respectively. Orlistat and pravastatin were also evaluated as a control, presenting free energy values of −5.6 and −6.2 kcal/mol for pancreatic lipase and HMGR, respectively. The most stabilized pose of the peptide bonds with PL and HMG-CoA reductase can be observed in Figure 6A,B, respectively. All the peptides identified in the different legume protein hydrolysates were able to interact with amino acid residues of PL and HMGR catalytic site. These peptides interacted with the enzymes mainly through hydrogen bonds and hydrophobic, polar and cation π interactions.

## 4. Discussion

Vicilin and legumin-like proteins comprise more than 70% of the total proteins found in the legumes used for this study. However, differences in the amino acid sequence of homologous proteins contained in the legume species used can lead to changes in the protein profile obtained after the hydrolysis treatment.

A higher DH is indicative of more peptide bonds cleaved, resulting in lower molecular weight peptides [38]. The application of the enzymatic hydrolysis with A and PP notably changed the structure of the legume native proteins. These changes can be noted in the disappearance of protein bands higher than 10 kDa after treatment and the formation of polypeptides with lower molecular mass. Similar findings were obtained in a study of legume isolate proteins [39].

The enzymatic action of pepsin and pancreatin in a sequential way to perform the simulated gastrointestinal digestion increases the enzyme selectivity and specificity, enabling the production of a larger number of small protein fractions or free amino acids [40]. Increasing cleavage sites are available for the second enzyme after the first one has acted, thus forming a more diverse mixture of amino acid residues in comparison to alcalase. According to these results, the action of the gastrointestinal enzymes was more effective to cleave distinct peptide bonds in black bean, chickpea and fava bean LPI. Even though enzymatic hydrolysis with PP treatment exhibited higher DH in the mentioned legumes, the action of alcalase showed the same effect as PP in green pea and lentil. These results suggest that fractions of vicilin commonly found in plants such as peas or lentils were susceptible to digestion with the different enzymatic treatments [41,42]. On the other hand, due to the very broad substrate specificity, alcalase can hydrolyze most peptide bonds within a protein molecule. Therefore, its use on substrates like legume proteins causes a high degree of hydrolysis, producing many peptides of small sizes [43]. The low DH values obtained in fava bean and chickpea LPH with alcalase could be related to vicilin, as this protein is known for its emulsifying, foaming and gelling properties [44,45]. Moreover, legumes rich in glutamic acid could have led to lower degradation by alcalase. This enzyme has a negative charge in physiological pH, which may diminish the DH due to the net negative charge present in glutamic acid [46]. In addition, resistance toward enzymatic activity may be a consequence of structural differences and the compactness of proteins [33]. Similar results were found by Ghribi et al. (2015) [47] in chickpea isolates hydrolyzed with alcalase, where it was found that a small degree of chickpea hydrolysis (DH = 4%) could enhance the emulsifying properties of chickpea protein. In another study performed with fava bean by Liu et al. (2019) [48], this moderate alcalase hydrolysis suggests the production of suitable lower molecular mass that could result in a more flexible peptide structure, increased surface charge and hydrophobicity, which could positively affect the emulsifying activity [41,49].

All the hydrolysates demonstrated the potential to inhibit pancreatic lipase and HMG-CoA reductase in a dose-dependent manner. According to the literature, Lee et al. (2015) [50] performed an inhibition assay for pancreatic lipase with phenolic compounds from methanolic extracts of seven selected legumes. The results showed pancreatic lipase inhibition in a dose-dependent manner, with no significant differences among red bean, chickpea, black soybean, yellow soybean and black-eyed pea extracts. The lowest reported IC_50_ values were for red bean (5.90 ± 0.59 mg/mL), chickpea (6.97 ± 2.19 mg/mL) and black soybean (6.65 ± 0.62 mg/mL). These values are higher (less potent) compared to most of the hydrolysates obtained in this work. The variation may be attributed to the differences in the extraction system, the varieties used and the bioactive compounds present in legume protein hydrolysates. Another report associated the peptide fragments released after protein enzymatic hydrolysis with the potential to act as HMGR inhibitors. For instance, the authors identified peptide sequences below 3 kDa from amaranth protein with hypocholesterolemic potential (GGV, IVG and VGVL) [51]. Recent studies performed on soybean glycinin and β-Conglycinin protein-derived peptides have also reported that these peptides could inhibit HMGR activity in vitro and in silico studies [52,53].

From all evaluated legume protein hydrolysate combinations used to block PL and HMG-CoA reductase activities, four combinations acted synergistically. PP–green pea with A–black bean, PP–green pea with A–green pea and A–black bean with A–green pea were demonstrated to inhibit more efficiently the activity of PL. Furthermore, the A–chickpea and PP–black bean combination acted synergistically against HMG-CoA reductase activity. Previous studies have demonstrated the efficacy of legume protein hydrolysates to inhibit markers related to obesity (black bean, green pea and chickpea hydrolysates) [19,54,55,56]. Several reports have demonstrated synergistic interactions among diverse bioactive compounds. For instance, synergistic and additive interactions of peptides produced from black bean for ACE inhibition were reported by Luna-Vital et al. (2015) [57]. The combination of peptides GLTSK and MTEEY showed a synergistic interaction, reducing the concentration needed to inhibit half of the enzymatic activity by approximately 30%. Further studies focusing on each peptide sequence are essential to confirm the possible effects of their interaction. These interactions can be significant and lead to lower effective dosages associated with the effects of pure compounds [58,59,60]. There is evidence that legume protein hydrolysates could interact with enzymes related to obesity. Exploring the possibilities to improve their performance, along with increasing the desired effect, is important.

The peptide profile of the protein hydrolysates obtained was different due to the legume source and enzyme used. As a result of the enzymatic hydrolysis and the specificity of the enzymes used, the biological activity of the legume protein hydrolysates was conferred by the specific combination of both protease used and source of proteins. The amino acid profile in peptide sequences with higher biological activity was associated with aliphatic amino acids, sulfur-containing and aromatic amino acids. Through molecular docking, it was possible to predict the binding affinity to the target enzymes (LP and HMG-CoA reductase). Peptide sequences presented lower predicted free energy values compared to the available drugs orlistat and pravastatin. These results suggest that they have a higher affinity for the catalytic site, thus exerting significant potential anti-adipogenesis activity. Non-polar interactions among peptide sequences and evaluated enzymes were more common due to the frequency of the aliphatic amino acids.

In addition to the effect of the enzymes related to lipid metabolism (LP and HMG-CoA reductase), obesity is associated with the production of reactive oxygen species and increased oxidative stress [8]. Peptides are able to contribute to the antioxidant defense in the body, being able to rapidly scavenge reactive oxygen species before cellular damage, therefore inactivating them [61]. The antioxidant capacity of the legume hydrolysates was measured using free radicals generated by ABTS and DPPH. Even though the meaning of the results obtained with these assays is limited as they use no physiological radicals, it is still possible to achieve representative data evaluating antioxidant activity [62]. Lentil protein hydrolysate generated with PP showed higher potential to scavenge the radical ABTS^•^ compared to the lentil protein hydrolysate generated using alcalase. Different legumes have demonstrated antioxidant capacities through this method. For instance, Ngoh et al. (2016) [63] determined the antioxidant activity presented in pinto bean peptides; in this study, peptide fractions < 3 kDa exhibited 42.2% inhibition of ABTS^•+^ scavenging activity. The results for DPPH^•^ suggest that lower DH in A-LPH may enhance the potential to scavenge the radical DPPH^•^. This is consistent with previous reports by Evangelho et al. (2017) [64], where it was observed that alcalase protein hydrolysates obtained from black bean showed higher DPPH^•^ scavenging capacities at lower DH. Likewise, similar findings were observed in a study performed by Kou et al. (2013) [65] showing that chickpea peptides obtained by alcalase exhibited 41.3% DPPH^•^ radical scavenging activity. Another free radical scavenging evaluation was performed with NO. This radical plays an important role in inflammatory processes; high levels of NO and its oxidized derivatives are known to be toxic, resulting in vascular damage and other ailments [66]. Legume protein hydrolysates showed the potential to scavenge NO, with important results. In the case of the NO scavenging capacity, the most potent legume hydrolysates were the ones hydrolyzed with PP. Pepsin/pancreatin protein hydrolysates presented a more extensive degree of hydrolysis, leading to more diversity of peptides in the protein hydrolysate. In a study performed by Oseguera-Toledo et al. (2015) [26], potent hydrolysate fractions of 5–10 kDa were obtained with the enzyme alcalase. These hydrolysates from black bean demonstrated the capacity to scavenge the radical NO with a range of inhibition from 57.46% to 68.26%.

Protein hydrolysates from selected legumes could participate in the inhibition of the enzymatic activity of LP and HMG-CoA reductase. Besides this, legume protein hydrolysates show the potential to exert antioxidant activity.

## 5. Conclusions

Legumes are an important source of ingredients for the formulation of healthy foods. Legume protein hydrolysates were able to block pancreatic lipase and HMG-CoA reductase using in silico and biochemical assays. Furthermore, legume protein hydrolysates showed important radical scavenging activities against ABTS, DPPH and NO radicals. These results shed light on the potential antioxidant activity of the peptides. More importantly, the combination of different legume protein hydrolysates inhibited synergistically the adipogenesis-related enzymes evaluated. Further studies are needed to determine the anti-obesity potential and the antioxidant capacity of pure synthesized peptides and their combinations. Mixtures of legume protein hydrolysates could be used as functional ingredients in the formulation of foods with the potential to prevent or treat non-communicable diseases such as obesity.

## Figures and Tables

**Table 1:** Physicochemical properties and their origin protein of the peptides obtained from the legume protein hydrolysates (LPH).

Sample	Molecular Mass (Da)	Peptide	Bioactive Sequence	pI	Net Charge	Hydrophobicity (kcal/mol)	Parental Protein ^1^
A–Black bean	526	PVALK	PV, VA, AL, LK	9.8	−1	15.2	Photosystem I P700 chlorophyll a apoprotein A2
	565	DYRL	DY, YR, RL	6.6	−2	13.0	Phaseolin alpha-type
	596	VEGHGV	VE, GH, GV	5.0	1	9.6	Protein kinase PVPK−1
	650	FEELN	EE, EL, LN	2.9	0	11.3	Putative resistance protein TIR 17
	751	THGPVGAN	TH, HG, GP, GPV, PV, VG, GA	7.3	0	10.2	Nitrate reductase
	757	ANGSPGGAGA	NG, GS, SP, PG, GG, GA, AG	5.6	0	16.1	Inositol-3-phosphate synthase
	834	KPSASCSR	KP, KPS, PS, AS	9.8	0	18.0	Glycerol-3-phosphate acyltransferase
	872	NVGPGSLET	NV, VG, VGP, GP, PG, GS, SL, ET	3.1	−1	13.8	Serine/threonine-protein phosphatase PP1
	983	PKEDLRLL	PK, KE, LR, LL	6.9	0	15.4	Phaseolin, alpha-type
	983	PSVADLRLL	PS, SV, VA, AD, LR, LL	6.7	0	13.8	DNA-directed RNA polymerase subunit beta
	1408	VNPDPAGGPTSGRAL	VN, VNP, NP, DP, PA, AG, GG, GP, PT, TS, SG, GR, RA, AL	6.7	2	14.5	Phaseolin, beta-type
PP–Black bean	656	DEGEAH	EG, GE, EA, AH	3.7	0	16.4	Phaseolin, alpha-type
	740	VELVGPK	VE, EL, LV, VG, VGP, GP, PK	6.5	−3	22.7	Phaseolin, beta-type
	742	VELTGPK	VE, LT, LTGP, TGP, TG, GP, PK	6.5	0	14.1	Phaseolin, alpha-type
	850	SGNGGGGGASM	SG, NG, GG, GA, AS	5.4	1	15.3	Glycine-rich cell wall structural protein
	855	SKPGGGSPVA	SK, KP, PG, GG, GS, SP, PV, VA	10.2	0	13.4	9-cis-epoxycarotenoid dioxygenase NCED1
	943	KPTTGKGALA	KP, PT, KPT, TT, TG, GK, GK, KG, AL, LA	10.6	2	16.1	9-cis-epoxycarotenoid dioxygenase NCED1
A–Green pea	539	GPAAGPA	GP, GPA, AA, AG, PA	5.6	1	13.29	Preprotein translocase subunit SECY
	541	TKGGAV	TK, KG, GG, GA, AV	10.1	0	11.98	Aminomethyltransferase, mitochondrial
	543	NPEGQ	NP, EG, GQ	3.1	0	5.7	Not reported
	544	TLSPGA	TL, TLS, LSP, SP, PG, GA	5.3	−2	12.3	Photosystem II CP43 reaction center protein
PP–Green pea	620	SPGDVF	SP, PG, GD, VF	3.0	0	10.8	Mitochondrial Type Ii Peroxiredoxin
	627	LTAVPAG	LT, TA, AV, AVP, VP, PA, AG	5.5	−1	14.4	ATP synthase subunit alpha
	678	HALLLL	HA, AL, LL, LLL	7.8	0	11.0	Photosystem II D2 protein
	683	SHLGAVT	SH, HL, LG, GA, AV, VT	7.5	0	9.1	Protein translocase subunit SecA, chloroplastic
	687	GRSAAGVA	GR, AA, AG, GV, VA	11.1	0	8.7	Asparagine synthetase, root
	780	HSLPGVAT	HS, SL, LP, LPG, PG, GV, VA, AT	7.5	−1	17.1	Dihydrolipoyl dehydrogenase, mitochondrial
	785	RDTAGLGP	TA, AG, GL, LG, LGP, GP	7.0	2	15.7	NAD(P)H-quinone oxidoreductase subunit 5
A–Chickpea	856	DLVLDVPS	LVL, LV, VL, VP, PS	2.7	0	15.2	Tubulin beta chain
	943	KPSSAAGAVR	KP, KPS, PS, AA, AG, GA, AV, VR	11.5	−1	11.1	Non-specific lipid-transfer protein
	1091	TAPHGGLPAGDV	TA, TAP, AP, PH, PHG, GG, GL, LP, PA, GD	4.9	1	13.5	Acidic endochitinase
PP–Chickpea	428	SPPE	SP, PP	3.1	−1	12.4	Not reported
	526	CSSSSG	SSS, SG	4.9	0	10.4	Alpha-amylase inhibitor
	620	SPGDV	SP, PG, GD	3.0	−1	11.1	Not reported
	812	TPSGLNPQ	TP, PS, SG, GL, LN, LNP, NP, PQ	5.2	1	8.5	Not reported
	812	TPEKNPQ	TP, EK, NP, PQ	6.5	0	10.8	Not reported
	815	EPNGGLVM	EP, PN, NG, GG, GL, LV, VM	3.0	0	10.4	Not reported
	900	HGAESAGGDT	HG, GA, AE, ES, AG, GG, GD	3.9	0	16.4	Non-specific lipid-transfer protein
	949	RTPVPPGLL	TP, PV, VP, VPP, PP, PPG, PG, PGL, GL, LL	11.1	−1	12.2	Acetyl-coenzyme A carboxylase carboxyl transferase subunit beta
A–Lentil	467	VVPGP	VV, VP, PG, PGP, GP	5.6	−1	10.6	Not reported
	533	PGDVF	PG, GD, VF	2.9	−2	12.9	Not reported
	596	DGHLR	DG, GH, HL, LR	7.5	0	15.5	Not reported
	652	EVGTFT	EV, VG, GT, TF, FT	3.0	1	13.8	Not reported
	678	FEDGLV	DG, DGL, GL, LV	2.9	−1	11.0	Not reported
	715	TPVSAGGK	TP, PV, VS, AG, GG, GK	9.8	0	8.4	Not reported
PP–Lentil	428	SPPE	SP, PP	3.1	−1	12.8	Not reported
	473	SPGDV	SP, PG, GD	3.0	0	8.6	Not reported
	552	VPPGAL	VP, VPP, PPG, PP, PG, GA, AL	5.6	1	12.6	Not reported
	627	LSVPGGV	SV, VP, PG, GG, GGV, GV	5.5	0	13.1	Not reported
	630	KGGLGVT	KG, GG, GL, LG, LGV, GV, VT	9.8	−1	13.6	Not reported
	758	TSPSPGDV	TS, SP, PS, PG, GD	3.0	−1	12.2	Not reported
	942	KTDVLPTGL	KT, TD, VL, VLP, LP, PT, TG, GL	6.7	0	8.1	Linoleate 9S-lipoxygenase
A–Fava bean	677	TPVHPQ	TP, PV, VH, HP, PQ	7.5	−1	15.2	Legumin type B alpha chain
	682	NLLAPR	NL, LL, LA, LAP, LLAP, AP, PR	10.7	1	8.7	Probable sucrose-phosphate synthase
	706	SFGGGGLL	SF, FG, GG, FGG, GL, LL	5.4	−1	18.3	14-3-3-like protein B
PP–Fava bean	715	FGGLLPL	FG, FGG, GG, GL, LL, LLP, LP, LPL, PL	5.4	0	11.0	NAD(P)H-quinone oxidoreductase subunit 5
	751	TKAGGTAF	TK, KA, AG, GG, GT, TA, AF	9.9	0	4.8	14-3-3-like protein B
	807	GPPVDVPQ	GP, GPP, PV, VD, VP, PQ	3.1	−1	12.9	Photosystem II protein D1
	810	PPNGPSEN	PP, PN, NG, GP, PS, SE	3.0	1	10.2	Acid beta-fructofuranosidase
	869	PPRSDSDP	PP, PR, DP	3.9	1	12.7	Not reported
	942	PYGVPVGVR	PY, YG, GV, VP, PV, VG, GV, VR	9.5	0	8.7	Elongation factor 1-alpha

^1^ As determined by BLAST^®^; A: enzymatic hydrolysis with alcalase; PP: enzymatic hydrolysis with pepsin/pancreatin; peptides obtained from the HPLC elution profile with intensity of at least 30% using LC–ESI–MS/MS, liquid chromatography–electrospray ionization tandem mass–spectrometry. Potential bioactivities were obtained from the BIOPEP database. Sequences were confirmed by BLAST tool, according to “UnitProtKB”. pI: isoelectric point; ACE: angiotensin-converting enzyme; DPP-IV: dipeptidyl peptidase IV; amino acid nomenclature: C, cysteine; H, histidine; I, isoleucine; M, methionine; S, serine; V, valine; A, alanine; G, glycine; L, leucine; P, proline; T, threonine; F, phenylalanine; R, arginine; Y, tyrosine; W, tryptophan; D, aspartic acid; N, asparagine; E, glutamic acid; Q, glutamine; K, lysine.

**Table 2:** Estimated free energy binding and chemical interactions among peptides, present in legumes hydrolyzed with alcalase and simulated gastrointestinal digestion, and the catalytic site of the pancreatic lipase and HMG-CoA reductase.

	Black Bean	Binding Affinity (kcal/mol)		Green Pea	Binding Affinity (kcal/mol)		Chickpea	Binding Affinity (kcal/mol)		Lentil	Binding Affinity (kcal/mol)		Fava bean	Binding Affinity (kcal/mol)	
		PL	HMG		PL	HMG		PL	HMG		PL	HMG		PL	HMG
**A**	FEELN	−6.1	−6.7	GPAAGPA	−7.0	−7.6	DLVLDVPS	−6.1	−6.8	VVPGP	−7.1	−7.4	TPVHPQ	−6.9	−7.6
	THGPVGAN	−6.3	−7.0	TKGGAV	−6.0	−6.8	KPSSAAGAVR	−6.1	−6.3	PGDVF	−7.0	−7.9	NLLAPR	−6.3	−7.6
	ANGSPGGAGA	−6.5	−8.1	NPEGQ	−6.3	−6.6	TAPHGGLPAGDV	−7.1	−7.7	DGHLR	−6.5	−6.9	SFGGGGLL	−5.8	−7.5
	KPSASCSR	−5.9	−6.7	TLSPGA	−6.3	−6.8				EVGTFT	−7.3	−8.2			
	NVGPGSLET	−6.9	−7.3							FEDGLV	−7.6	−7.0			
	PKEDLRLL	−5.6	−7.4							TPVSAGGK	−6.0	−6.2			
	PSVADLRLL	−5.9	−7.7				SPPE	−6.6	−7.7						
	VNPDPAGGPTSGRAL	−7.0	−8.0	SPGDVF	−6.7	−7.3	CSSSSG	−5.5	−6.1	SPPE	−6.8	−7.5			
**PP**	DEGEAH	−6.3	−7.6	LTAVPAG	−6.3	−6.3	SPGDV	−7.0	−6.8	SPGDV	−6.4	−6.6	FGGLLPL	−6.6	−7.6
	VELVGPK	−6.2	−6.9	HALLLL	−5.9	−6.9	TPSGLNPQ	−6.9	−7.8	VPPGAL	−6.4	−7.7	TKAGGTAF	−5.5	−7.2
	VELTGPK	−6.4	−6.7	SHLGAVT	−6.5	−6.7	TPEKNPQ	−6.9	−7.1	LSVPGGV	−6.2	−7.2	GPPVDVPQ	−5.8	−6.1
	SGNGGGGGASM	−6.0	−6.8	GRSAAGVA	−5.8	−6.7	EPNGGLVM	−5.7	−7.0	KGGLGVT	−6.4	−6.7	PPNGPSEN	−5.9	−8.1
	SKPGGGSPVA	−5.6	−7.9	HSLPGVAT	−6.8	−7.1	HGAESAGGDT	−5.8	−6.8	TSPSPGDV	−7.0	−7.4	PPRSDSDP	−7	−7.7
	KPTTGKGALA	−6.4	−7.0	RDTAGLGP	−6.5	−7.3	RTPVPPGLL	−6.8	−7.9	KTDVLPTGL	−6.3	−6.7	PYGVPVGVR	−6.9	−8.8

A: enzymatic hydrolysis with alcalase; PP: enzymatic hydrolysis with pepsin/pancreatin; PL: pancreatic lipase; HMG: HMG-CoA reductase. Amino acid nomenclature: C, cysteine; H, histidine; I, isoleucine; M, methionine; S, serine; V, valine; A, alanine; G, glycine; L, leucine; P, proline; T, threonine; F, phenylalanine; R, arginine; Y, tyrosine; W, tryptophan; D, aspartic acid; N, asparagine; E, glutamic acid; Q, glutamine; K, lysine.

## Abbreviations

A: alcalase; A-LPH: legume protein hydrolyzed with alcalase; ABTS: 2,2′-azinobis (3-ethyl-benzothiazoline-6-sulfonic acid) diammonium salt; ACE: angiotensin-converting enzyme; DH: degree of hydrolysis; DPP-IV: dipeptidyl peptidase-IV; DPPH: 2,2-diphenyl-1-picrylhydrazyl; FDA: U.S. Food and Drug Administration; HMGR: 3-hydroxy-3-methylglutaryl coenzyme A reductase; LC–ESI–MS/MS: liquid chromatography–electrospray ionization–mass spectrometry; LPH: legume protein hydrolysates; LPI: legume protein isolates; MOPS: 3-Morpholinopropane-1-sulfonic acid; NaTDC: sodium taurodeoxycholate hydrate; NO: nitric oxide; PL: pancreatic lipase; PP: pepsin and pancreatin; PP-LPH: legume protein hydrolyzed with pepsin/pancreatin; SDS–PAGE: sodium dodecyl sulfate–polyacrylamide gel electrophoresis: SNP: sodium nitroprusside; TE: Trolox equivalent; TNBS: trinitrobenzene sulfonic acid.
